# Relationship between body roundness index and mortality rates and life expectancy in populations with metabolic syndrome

**DOI:** 10.3389/fcvm.2025.1608436

**Published:** 2026-01-05

**Authors:** Xicheng Wang, Taisheng Wu, Yan Hong

**Affiliations:** Department of Cardiology, Huaihe Hospital of Henan University, Kaifeng, Henan, China

**Keywords:** all-cause mortality, body roundness index, cardiovascular mortality, life expectancy, metabolic syndrome

## Abstract

**Background:**

Cardiovascular Disease (CVD) represents a significant global public health challenge. Metabolic Syndrome (MetS) notably elevates the risk of CVD, with MetS patients facing approximately 2–3 times the risk compared to the general population. Body Roundness Index (BRI), a novel anthropometric marker, has recently gained attention; However, its comprehensive association with mortality risk in MetS populations remains incompletely elucidated.

**Methods:**

This longitudinal study analyzed data from 10 consecutive cycles of the National Health and Nutrition Examination Survey (NHANES) between 1999 and 2018, comprising 19,535 participants with MetS. We employed Cox proportional hazards regression models to elucidate the association between BRI and mortality risk. Nonlinear relationships were comprehensively examined using smooth curve fitting and two-segment Cox regression models to identify potential threshold effects. Life table analysis was utilized to calculate life expectancy by levels of the BRI, with multiple sensitivity analyses performed to validate the robustness and consistency of the primary findings.

**Results:**

BRI demonstrated a U-shaped association with mortality risk (*P* for nonlinearity <0.001), with the highest quintiles showing markedly increased all-cause (HR: 1.29, 95% CI: 1.12–1.48) and cardiovascular mortality risk (HR: 1.39, 95% CI: 1.13–1.73). BRI and BMI performed similarly in predicting all-cause mortality (AUC: 0.810 vs. 0.802, *P* = 0.18). Gender-stratified analyses revealed significant effect modification (*P* < 0.001): males in Q5 exhibited a 56% increased mortality risk (HR: 1.56, 95% CI: 1.30–1.87) and a 3.8-year reduction in life expectancy at age 45, while females in Q4 showed a protective effect (HR: 0.77, 95% CI: 0.66–0.90) and females in Q5 demonstrated only a modest 0.9-year life expectancy reduction.

**Conclusion:**

Our findings demonstrate a U-shaped association between BRI and all-cause and cardiovascular mortality in individuals with MetS. Notably, BRI showed comparable predictive performance to BMI for all-cause mortality in this population. Maintaining an optimal BRI level may be associated with improved survival outcomes.

## 1 Introduction

Cardiovascular disease (CVD) remains the leading global cause of death and disease burden (1), with obesity as a central risk factor that amplifies CVD mortality through mechanisms including hypertension, dyslipidemia, and glucose metabolism disorders (2). This risk is substantially magnified in metabolic syndrome (MetS), where excess visceral fat triggers chronic inflammation, insulin resistance, and endothelial dysfunction, creating a pathological cascade that increases cardiovascular mortality 2–3-fold compared to the general population (3–11). Traditional anthropometric measures such as BMI and waist circumference inadequately capture fat distribution heterogeneity, with BMI explaining less than 15% of cardiovascular death risk and failing to quantify visceral adiposity—the key driver of metabolic complications (12, 13).

The Body Roundness Index (BRI), a novel marker integrating height and waist circumference, offers superior quantification of abdominal fat distribution and has demonstrated enhanced predictive value for mortality across diverse populations, including patients with diabetes, MetS, and heart failure (14–19). However, while associations between BRI and mortality risk have been established, translating these relative risks into clinically intuitive metrics such as life expectancy years lost remains unaddressed. Life expectancy analysis provides an absolute measure of disease burden that is more meaningful for patient communication, clinical decision-making, and public health policy than hazard ratios alone. For MetS patients requiring lifelong risk management, quantifying the impact of adiposity on years of life lost offers a powerful tool for risk stratification and motivating interventions. Despite its clinical importance, no prior studies have investigated the relationship between BRI and life expectancy in MetS populations.

Leveraging a comprehensive longitudinal cohort from NHANES 1999–2018, this study bridges this gap by examining the association between BRI and life expectancy among MetS patients, providing novel insights for personalized risk assessment and precision treatment strategies.

## 2 Materials and methods

### 2.1 Source and participant selection

This investigation focused on the longitudinal epidemiological data from NHANES, which was conducted in the U.S. from 1999 through 2018. NHANES, which has received approval from the NCHS Ethical Review Board (ERB), systematically collects health-related data from a stratified sample that represents the non-institutionalized population of the United States, a design for sampling that uses multiple stages of probability. Written consent was obtained from all participants after informing them. For a comprehensive overview of the NHANES database, visit the website (https://www.cdc.gov/nchs/nhanes/). Among 101,316 initially screened participants, we sequentially excluded: those with unreliable death status (*n* = 42,252) or did not complete both the household interview and the mobile examination center (MEC) health examination (*n* = 2,811); individuals aged <20 years (*n* = 3,966); those with missing BRI information (*n* = 3,156); and participants not meeting metabolic syndrome criteria (*n* = 29,596). The final analysis included 19,535 participants (Supplementary Figure 1). Specifically, participants with unreliable mortality status were defined as those not eligible for mortality linkage (ELIGSTAT ≠ 1) according to NCHS protocol.

### 2.2 Explanation of MetS

According to NCEP ATP III standards, MetS is identified when three or more of five particular conditions are present: (1) Central obesity (waist circumference ≥102 cm for men and ≥88 cm for women); (2) Hypertriglyceridemia [serum triglycerides ≥150 mg/dL (1.7 mmol/L) or on specific drug treatment]; (3) Low HDL cholesterol (HDL-C) (HDL-C <40 mg/dL for men and <50 mg/dL for women); (4) High arterial pressure (systolic blood pressure ≥130 mmHg or diastolic blood pressure ≥85 mmHg, or on antihypertensive drug treatment); (5) High blood glucose (fasting plasma glucose ≥100 mg/dL or on hypoglycemic drug treatment ) (1).

The arithmetic mean of up to four consecutive blood pressure readings, taken by certified inspectors following a standardized protocol, was used to calculate the values (2). Information on medication use and past medical history was obtained through structured interviews conducted by trained personnel.

### 2.3 BRI assessment and covariates

The BRI was determined using this formula: $\mathrm{BRI}=364.2\text{-}365.5\times \sqrt{1-\left(\frac{{\left(\frac{\mathrm{WC}(m)}{2\pi }\right)}^{2}}{(0.5\times \mathrm{Height}(m){)}^{2}}\right)}$ (3), where waist circumference (WC) and height are measured in centimeters following a standardized protocol by trained health technicians.

Demographic characteristics, including age, sex (male, female), race/ethnicity (Non-Hispanic White, Non-Hispanic Black, Other), educational attainment (low, medium, high), income level (low, medium, high), and marital status (married/living with partner, other) were collected through structured interviews. Lifestyle factors were assessed using standardized questionnaires, encompassing smoking status (never, former, or current), alcohol consumption (none, moderate, or heavy), and leisure-time physical activity. Dietary quality was evaluated using the Healthy Eating Index-2015 (HEI-2015) score derived from 24-h dietary recall data.

### 2.4 Mortality ascertainment

Linking with the National Death Index (NDI) was used to determine mortality status, with follow-up continuing until December 31, 2019. Cardiovascular deaths were recognized using the international system used to classify diseases, Tenth Edition, codes I00 to I99. The time for follow-up was calculated starting from the interview date and ending at the date of death or the follow-up's conclusion.

### 2.5 Statistical analysis

All analyses accounted for NHANES's complex survey design using MEC examination weights (WTMEC2YR/4YR), strata, and clustering variables. All findings were adjusted to offer estimates that represent the non-institutionalized US civilian population on a national level. Means and standard deviations (SD) were utilized to express continuous variables, and frequencies and percentages were used for categorical variables. Group differences for categorical and continuous variables were evaluated using chi-square and Kruskal–Wallis tests, respectively. Multiple imputation was used for missing data (<5%) with five imputations performed using the Fully Conditional Specification (FCS) method. For all-cause mortality, a Cox proportional hazards model was used to examine the association between BRI and mortality outcomes; for cardiovascular mortality, given that other causes of death act as competing events, the Fine-Gray subdistribution hazard model was applied to provide more accurate risk estimates. Models were adjusted stepwise: Crude Model (unadjusted), Model 1 (adjusted for sex and age as a continuous variable), and Model 2 [further adjusted for race/ethnicity, drinking status, education, smoking status, family income-poverty ratio, family history of diabetes, marital status, HEI-2015 score (continuous), leisure-time physical activity (continuous), and other comorbidities (CVD, hyperuricemia)). ROC curves were used to compare the advantage of BRI over BMI in predicting mortality outcomes. A curve fitting analysis was used to assess the nonlinear connection between BRI and mortality outcomes. The relationship between BRI and mortality risk was first visualized with a smooth curve. Subsequently, the study used a two-section Cox proportional hazards regression model to explore potential nonlinear associations, allowing the slope to change at a specific threshold point. The optimal threshold was determined using a pointwise approach, selecting the value within the BRI distribution that maximized the log-likelihood function. The comparison between linear and piecewise linear models in depicting the BRI-mortality link was conducted using the log-likelihood ratio test. The adjusted hazard ratios and their 95% confidence intervals were computed for each side of the established threshold.

Life expectancy was calculated using the life table method, extrapolating mortality rates for those aged 80 and above using an exponential growth model (with an annual growth rate of 12%–15%). Smoothing was applied using the LOESS method, and 95% confidence intervals were computed through 1,000 Bootstrap repetitions. Life expectancy was calculated at ages 45, 55, and 65. Sensitivity analyses included: (1) excluding participants who died in the first two years of follow-up helps to mitigate reverse causation bias; (2) excluding participants with CVD or cancer at baseline; and (3) further adjusting for the NHANES survey cycle. The proportional hazards assumption was verified using Schoenfeld residuals and log-log survival plots. All statistical analyses were performed using EmpowerRCH and SAS 9.4 software (version 9.4; SAS Institute, Cary, NC). Statistical significance was set at a two-sided *P*-value <0.05.

## 3 Results

### 3.1 Baseline characteristics of study participants

Based on NHANES 1999–2018 cohort study data (*n* = 19,535), this study analyzed the relationship between quintiles of BRI (Table 1). The findings revealed several key trends in population characteristics as BRI levels increase. Demographically, the proportion of famales gradually increased (from 46.67% in Q1 to 64.74% in Q5), and non-Hispanic whites dominated across all groups (ranging from 68.45% to 71.62%). The majority of the population was under 65 years old (ranging from 69.51% to 76.31%). Physical activity time decreased significantly across BRI quintiles (from 212.01 min in Q1 to 100.78 min in Q5), and poorer diet quality was observed (the proportion of low HEI-2015 scores increased from 67.60% in Q1 to 78.28% in Q5). The proportion of smokers and heavy drinkers decreased with increasing BRI. The incidence of comorbidities, including CVD (from 11.93% in Q1 to 14.78% in Q5), hyperuricemia (from 20.08% in Q1 to 38.97% in Q5), and hypertension (from 81.63% in Q1 to 85.70% in Q5), showed an upward trend. In general, the group with the highest BRI had a higher proportion of females, along with lower levels of physical activity, poorer diet quality, and a higher incidence of cardiometabolic diseases.

**Table 1:** Baseline characteristics of participants with BRI quintiles.

Weighted no. (%), millions					
Characteristic	Q1	Q2	Q3	Q4	Q5
Prevalence	16.02 (21.44)	15.24 (20.40)	14.42 (19.29)	14.38 (19.25)	14.66 (19.63)
Participants, No.	3,908	3,906	3,907	3,907	3,907
leisure time	212.01 (9.56)	191.35 (10.12)	166.54 (7.90)	132.72 (6.96)	100.78 (5.58)
Sex					
Female	7.48 (46.67)	6.94 (45.57)	6.82 (47.34)	8.15 (56.68)	9.49 (64.74)
Male	8.54 (53.33)	8.29 (54.43)	7.59 (52.66)	6.23 (43.32)	5.17 (35.26)
Race and ethnicity					
Non-Hispanic White	11.47 (71.62)	10.79 (70.81)	10.06 (69.77)	10.04 (69.82)	10.04 (68.45)
Non-Hispanic Black	1.66 (10.34)	1.47 (9.61)	1.45 (10.06)	1.65 (11.49)	2.06 (14.04)
Other	2.89 (18.04)	2.98 (19.57)	2.91 (20.18)	2.69 (18.69)	2.57 (17.51)
Age					
≤65	12.06 (75.27)	11.11 (72.90)	10.02 (69.51)	10.08 (70.07)	11.19 (76.31)
>65	3.96 (24.73)	4.13 (27.10)	4.39 (30.49)	4.30 (29.93)	3.47 (23.69)
Family history of DM					
No	9.51 (59.37)	8.83 (57.92)	8.06 (55.88)	7.28 (50.60)	6.82 (46.51)
Yes	6.51 (40.63)	6.41 (42.08)	6.36 (44.12)	7.10 (49.40)	7.84 (53.49)
Education level					
Less than high school	2.76 (17.25)	2.84 (18.66)	3.22 (22.34)	3.12 (21.73)	2.97 (20.27)
High school or equivalent	4.15 (25.93)	3.96 (25.99)	3.72 (25.80)	3.77 (26.21)	4.31 (29.39)
College Graduate or above	9.09 (56.81)	8.43 (55.35)	7.47 (51.86)	7.49 (52.07)	7.38 (50.33)
Income					
Low	2.72 (18.40)	2.77 (19.55)	2.90 (21.83)	3.09 (23.24)	3.82 (27.95)
Medium	5.36 (36.27)	5.16 (36.41)	4.99 (37.54)	5.20 (39.14)	5.40 (39.59)
High	6.70 (45.33)	6.23 (44.04)	5.40 (40.63)	5.00 (37.62)	4.43 (32.46)
Smoking					
Never smoke	7.91 (49.36)	7.61 (50.01)	7.05 (48.94)	7.17 (49.89)	7.43 (50.65)
Ever smoke	4.25 (26.53)	4.83 (31.71)	4.70 (32.65)	4.67 (32.51)	4.57 (31.19)
Current smoke	3.86 (24.10)	2.78 (18.28)	2.65 (18.42)	2.53 (17.60)	2.66 (18.16)
Drinking status					
No drinking	5.25 (37.53)	5.30 (39.64)	5.59 (44.79)	6.12 (49.18)	6.59 (52.80)
Light drinking	7.31 (52.22)	6.89 (51.56)	5.87 (47.01)	5.38 (43.23)	5.22 (41.80)
Heavy drinking	1.43 (10.25)	1.18 (8.80)	1.02 (8.20)	0.94 (7.59)	0.67 (5.40)
CVD	1.91 (11.93)	1.70 (11.14)	1.84 (12.76)	2.02 (14.03)	2.17 (14.78)
Hypertension	13.08 (81.63)	11.97 (78.52)	11.64 (80.77)	11.80 (82.05)	12.57 (85.70)
Hyperlipidemia	15.71 (98.08)	14.69 (96.39)	13.83 (95.93)	13.65 (94.94)	13.98 (95.36)
Hyperuricemia	3.22 (20.08)	3.68 (24.14)	3.87 (26.88)	4.73 (32.86)	5.71 (38.97)
Marital status					
Married/Living with partner	10.68 (67.59)	10.47 (69.30)	9.69 (67.66)	9.04 (63.49)	8.63 (59.28)
other	5.12 (32.41)	4.64 (30.70)	4.63 (32.34)	5.20 (36.51)	5.93 (40.72)
Dietary quality, HEI-2015 score, *n* (%)					
<60	10.83 (67.60)	10.98 (72.05)	10.52 (72.95)	10.55 (73.37)	11.48 (78.28)
≥60	5.19 (32.40)	4.26 (27.95)	3.90 (27.05)	3.83 (26.63)	3.19 (21.72)

### 3.2 Associations between body roundness index and mortality risk

In the continuous variable analysis, Model 2, after full adjustments, demonstrated that each additional unit of BRI was tied to a 5% increased likelihood of dying from any cause (HR: 1.05, 95% CI: 1.03–1.07, *P* < 0.0001), a 9% elevation in the risk of mortality due to cardiovascular reasons was observed (HR: 1.09, 95% CI: 1.05–1.14, *P* < 0.0001). In the quintile analysis, using Q3 as the reference group and after adjusting for demographics, lifestyle factors, and comorbidities (Model 2), The highest BRI category (Q5) was associated with a substantially increased risk of mortality from all causes (HR: 1.29, 95% CI: 1.12–1.48, *P* = 0.0004). No significant association was found between the other quintiles (Q1, Q2, Q4) risk of death from any source. Concerning deaths related to heart disease, Model 2 indicated a substantial rise in risk for the Q5 group (HR: 1.39, 95% CI: 1.13–1.73), while no significant differences were observed in the other quintiles (Table 2). This association persisted after adjusting for gender, age, race/ethnicity, education level, income-to-poverty ratio, drinking status, smoking status, family history of diabetes, marital status, HEI-2015 score, leisure-time physical activity, and other comorbidities (CVD, hyperuricemia). These findings suggest a significant positive correlation between BRI and the risk of all-cause and cardiovascular mortality, independent of traditional risk factors. Sensitivity analyses were performed without including those who died in the first two years after the follow-up began, those with pre-existing CVD or cancer at baseline, further adjustments for NHANES survey cycles confirmed consistent results concerning the correlation between BRI and mortality rates due to all causes and cardiovascular conditions (Supplementary Tables 1, 2, and 3).

**Table 2:** Hazard ratios of BRI for mortality risk in metabolic syndrome.

All-cause mortality	Event (per 1,000 person-years)	Crude Model HR (95% CI)^a^	*P*	Model 1 HR (95% CI)^a^	*P*	Model 2 HR (95% CI)^a^	*P*
BRI		1.03 (1.01,1.05)	0.003	1.08 (1.05,1.10)	<0.001	1.05 (1.03,1.07)	<0.0001
BRI (quintiles)							
Q1 (1.19–5.02)	855 (26.70)	0.84 (0.75–0.94)	<0.01	0.97 (0.87–1.09)	0.58	1.01 (0.90–1.14)	0.84
Q2 (5.02–5.88)	794 (24.60)	0.79 (0.69–0.90)	<0.001	0.85 (0.75–0.96)	0.01	0.89 (0.79–1.02)	0.1
Q3 (5.88–6.81)	870 (28.16)	1 (reference)		1 (reference)		1 (reference)	
Q4 (6.81–8.22)	833 (27.43)	0.94 (0.84–1.05)	0.26	1.01 (0.90–1.14)	0.84	0.98 (0.87–1.11)	0.77
Q5 (8.22–23.48)	753 (25.70)	1.06 (0.92–1.21)	0.44	1.42 (1.24–1.63)	<0.001	1.29 (1.12–1.48)	0.001
*P* for trend			<0.001		<0.001		<0.001
CVD mortality							
BRI		1.06 (1.03,1.09)	<0.001	1.13 (1.09,1.18)	<0.001	1.09 (1.05,1.14)	<0.001
BRI (quintiles)							
Q1 (1.19–5.02)	229 (7.15)	0.76 (0.63–0.93)	0.01	0.89 (0.73–1.08)	0.24	0.93 (0.75–1.14)	0.46
Q2 (5.02–5.88)	203 (6.29)	0.76 (0.61–0.94)	0.01	0.82 (0.67–1.00)	0.05	0.87 (0.70–1.08)	0.21
Q3 (5.88–6.81)	246 (7.96)	1 (reference)		1 (reference)		1 (reference)	
Q4 (6.81–8.22)	231 (7.61)	0.99 (0.81–1.22)	0.95	1.09 (0.89–1.34)	0.39	1.04 (0.84–1.27)	0.74
Q5 (8.22–23.48)	218 (7.44)	1.17 (0.96–1.43)	0.12	1.68 (1.36–2.07)	<0.001	1.39 (1.13–1.73)	0.002
*P* for trend		<0.001			<0.001		<0.001

Crude Model: Unadjusted results. Model 1: Results adjusted for age and sex; Model 2: Results adjusted for sex, age (continuous), race/ethnicity, education, family income-poverty ratio, drinking status, smoking status, family history of diabetes, marital status, HEI-2015 score (continuous), leisure-time physical activity (continuous), and other comorbidities (CVD, hyperuricemia). a sampling weights were considered in analyses.

### 3.3 Exploring nonlinear relationships

The analysis using a Cox regression model consisting of two sections revealed notable nonlinear associations between BRI and the likelihood of dying from any cause or cardiovascular diseases (Table 3; Supplementary Figure 2). We identified clinically relevant BRI thresholds of 5.13 for all-cause mortality and 5.56 for cardiovascular mortality. For all-cause mortality, when BRI was less than 5.13, each additional unit of BRI was linked to a 19% lower risk (HR: 0.81, 95% CI: 0.75–0.87, *P* < 0.0001). However, when BRI was greater than or equal to 5.13, for every 1-unit rise in BRI, there was a 7% higher risk (HR: 1.07, 95% CI: 1.04–1.09, *P* < 0.0001). Regarding cardiovascular mortality, when BRI was less than 5.56, each additional unit of BRI corresponded to a 12% lower risk (HR: 0.88, 95% CI: 0.78–0.99, *P* = 0.03). When BRI was greater than or equal to 5.56, an increase of 1 unit in BRI corresponded to an 8% elevation in risk (HR: 1.08, 95% CI: 1.04–1.13, *P* = 0.0001). The log-likelihood ratio test confirmed the statistical significance of these nonlinear associations (both *P* < 0.01), implying a U-shaped link where both low and high BRI levels could raise the likelihood of death. Notably, these threshold effects exhibited significant gender differences: males in Q5 (BRI >threshold) experienced a 56% mortality increase and 3.8-year life expectancy loss, while females showed a protective effect at Q4 (HR: 0.77) with only modest impact at Q5 (0.9-year loss). We evaluated the discriminative ability of both BRI and BMI for predicting all-cause mortality. The area under the ROC curve (AUC) was 0.810 for BRI and 0.802 for BMI, with no statistically significant difference (*P* = 0.18) (Supplementary Figure 3).

**Table 3:** The results of two-piecewise cox regression model between BRI and mortality.

BRI group	Adjusted HR (95%CI)	*P* value
All-cause mortality		
Inflection point	5.13	
BRI <5.13	0.81 (0.75–0.87)	<0.0001
BRI ≥5.13	1.07 (1.04–1.09)	<0.0001
*P* for log likelihood ratio test		<0.001
CVD mortality		
Inflection point	5.56	
BRI <5.56	0.88 (0.78–0.99)	0.03
BRI ≥5.56	1.08 (1.04–1.13)	0.0001
*P* for log likelihood ratio test		0.004

The model was adjusted for sex, age (continuous), race/ethnicity, education, family income-poverty ratio, drinking status, smoking status, family history of diabetes, marital status, HEI-2015 score (continuous), leisure-time physical activity (continuous), and other comorbidities (CVD, hyperuricemia).

### 3.4 Subgroup analyses

**Figure 1:**
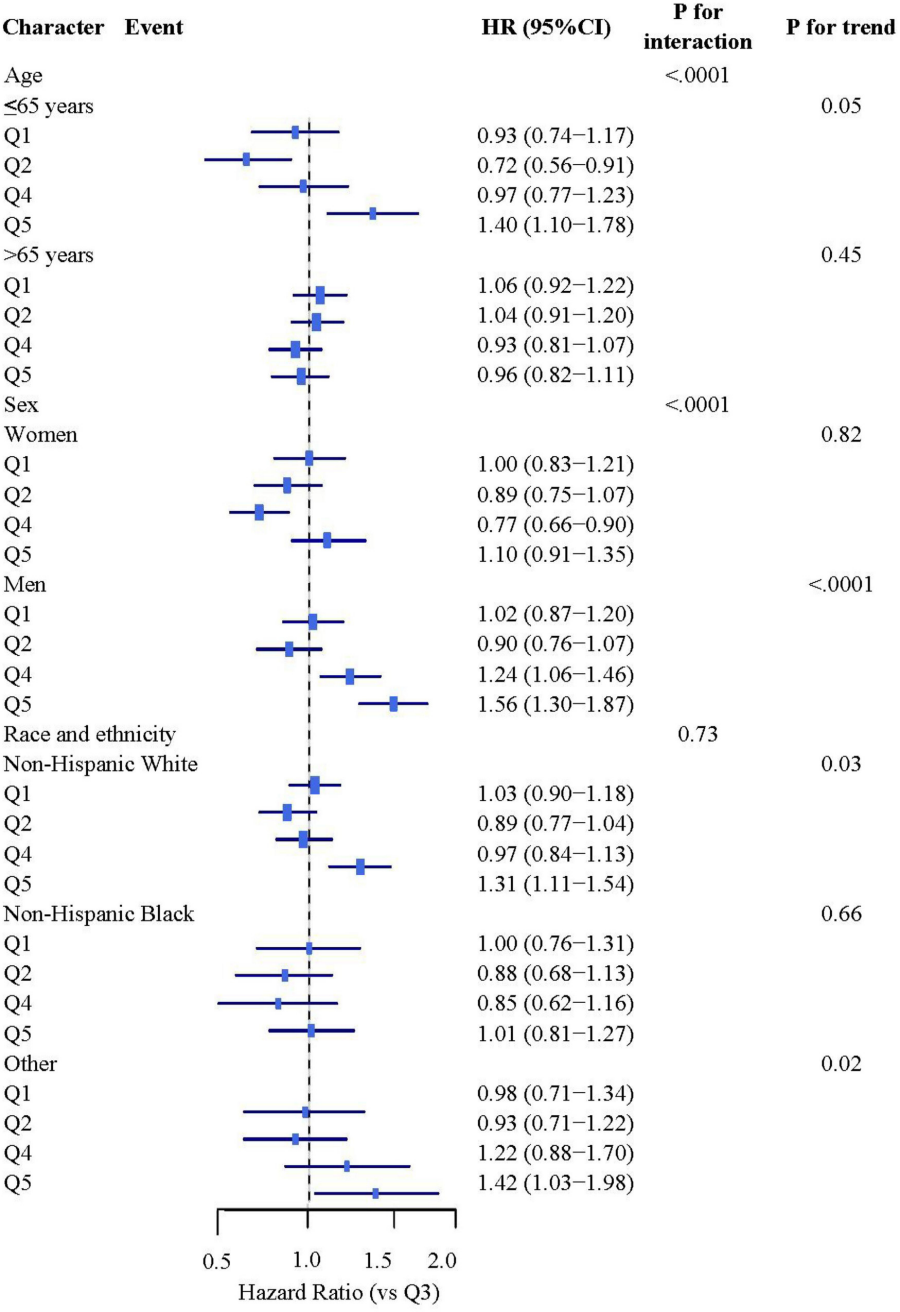
Stratified analysis study on the association between BRI and mortality rates. Stratified analyses were adjusted for covariates including sex, age (continuous), race/ethnicity, education, family income-poverty ratio, drinking status, smoking status, family history of diabetes, marital status, HEI-2015 score (continuous), leisure-time physical activity (continuous), and other comorbidities (cardiovascular disease, hyperuricemia).

Subgroup analyses revealed significant effect modification by age and sex (both *P* < 0.001, Figure 1). Among participants ≤65 years, Q5 was associated with a 40% increased mortality risk (HR: 1.40, 95% CI: 1.10–1.78), while no significant association was observed in those >65 years. Sex-stratified analyses showed a pronounced risk increase in males with Q5 (HR: 1.56, 95% CI: 1.30–1.87), whereas females showed a protective effect in Q4 (HR: 0.77, 95% CI: 0.66–0.90). No significant interactions were observed for race or other variables (all *P* > 0.05, Supplementary Figure 4).

### 3.5 Relationship between BRI and life expectancy

**Figure 2:**
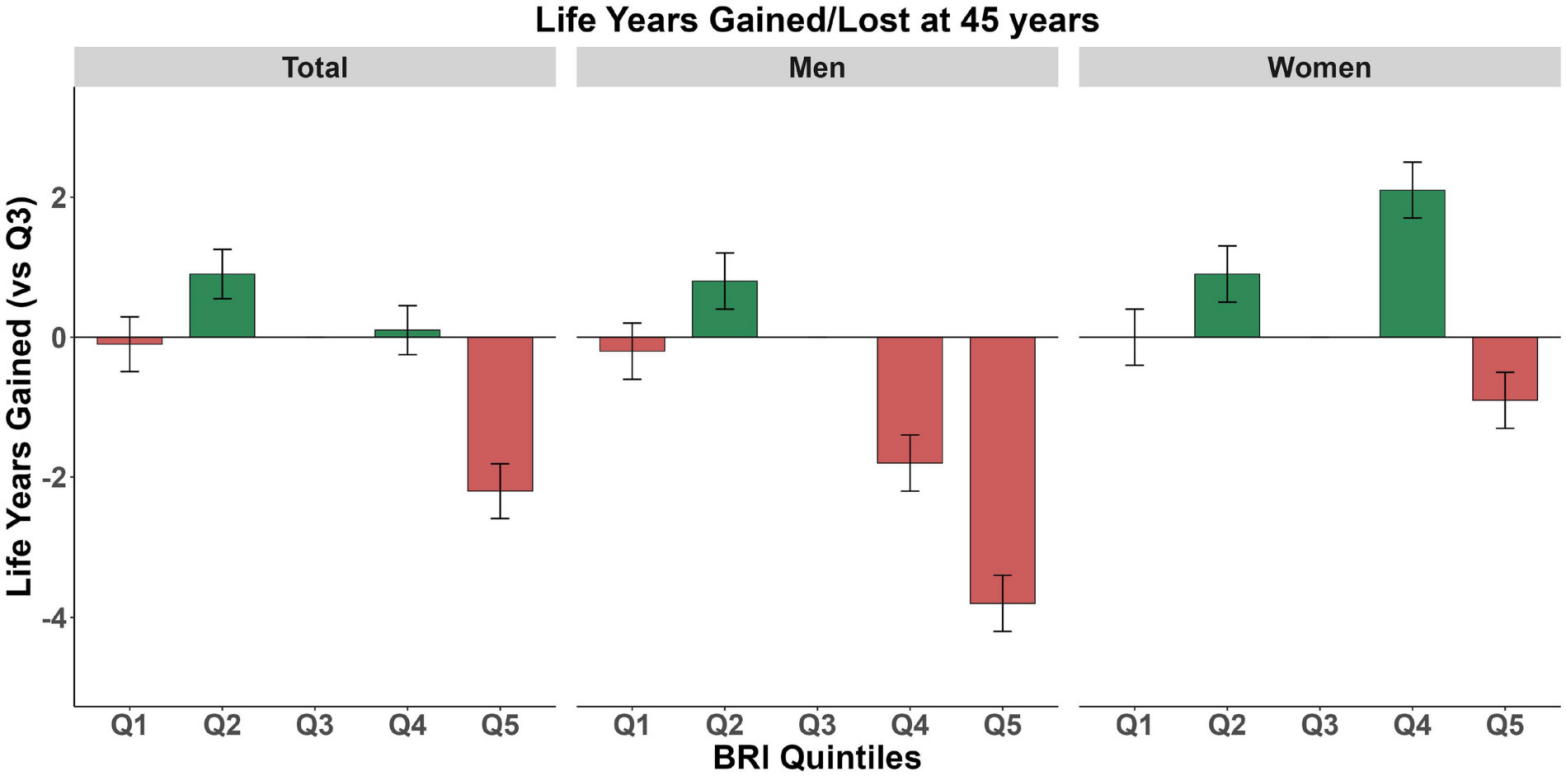
Expected lifespan at age 45: stratified analysis by body roundness index in total population, women, and men. Green indicates lifespan gain, while red indicates lifespan loss.

There were significant differences in life expectancy at age 45 across different quintiles of BRI (Figure 2). In the overall population, compared to Q3 (the reference group), life expectancy increased by 0.9 years (95% CI: 0.55, 1.25) in Q2 and decreased by 2.2 years (95% CI: −2.59, −1.81) in Q5. Stratified analysis by gender revealed a distinct gradient among men from Q2 to Q5: an increase of 0.8 years (95% CI: 0.4, 1.2) in Q2 and decreases of 1.8 years (95% CI: −2.2, −1.4) and 3.8 years (95% CI: −4.2, −3.4) in Q4 and Q5, respectively. Women showed a different pattern: the largest increase was observed in Q4, reaching 2.1 years (95% CI: 1.7, 2.5), while Q5 only showed a decrease of 0.9 years (95% CI: −1.3, −0.5). This suggests that the association between BRI level at age 45 and life expectancy is gender-specific, with a more pronounced negative impact of high BRI levels on men's life expectancy. Furthermore, we estimated life expectancy and years of life gained across different BRI strata at ages 55 and 65 (Supplementary Figures 5, 6).

## 4 Discussion

Based on the large-scale cohort data from NHANES 1999 to 2018, this study delved into the association between the BRI and death from any cause as well as life expectancy among patients with MetS, revealing several significant findings. Firstly, Our study verified Shaped like a U link concerning BRI and risk of death, with turning points for deaths from any cause and those specifically from cardiovascular diseases at 5.13 and 5.56, respectively. This result is similar to the inflection points observed in a diabetic population (5.54 and 5.21) (20), but higher than those observed in the general population (3.9) (16), and lower than those found in a hypertensive population (5.9 and 6.3) (21). These disease-specific thresholds suggest that BRI cutoffs for screening and intervention may need tailoring based on underlying metabolic conditions, offering a practical tool for risk stratification in clinical practice. This suggests that different disease states may affect the optimal range of BRI, which is also evident in a study conducted among osteoporotic Americans (22). The U-shaped correlation indicates that both excessively high and low body fat distribution may elevate the risk of death. This aligns with two studies observed among Chinese populations (23, 24). In recent years, multiple studies have further corroborated the predictive value of BRI in various metabolic-related diseases. Research has shown that BRI acts as an independent indicator of death risk in the group of patients with fatty liver disease connected to metabolic dysfunction (MAFLD), reflecting its potential in evaluating the prognosis of liver metabolic diseases (25). A study conducted among Jordanian adults found that BRI is the best indicator for predicting MetS, surpassing traditional anthropometric indicators (26). Additionally, BRI has been proven to be the most powerful indicator for distinguishing between hypertensive and normotensive patients, it is seen as the most effective predictor and standalone determinant of diabetes development in those with high blood pressure (18, 27). These findings are consistent with our research, further endorsing the clinical application of BRI as a risk assessment instrument for cardiometabolic diseases.

A notable finding of this study is that in the metabolic syndrome cohort, the predictive efficacy of BRI for all-cause mortality was not significantly superior to BMI. Previous studies in chronic kidney disease populations have reported that BRI outperforms BMI, but our results indicate that in individuals with established metabolic syndrome, the complexity of BRI does not provide additional benefit. This does not diminish the value of BRI—rather, it clarifies that in specific populations, simpler metrics may be sufficient, and the choice of adiposity index should be population-specific.

Secondly, for the first time, we quantified the relationship between BRI and life expectancy among the MetS population. Compared to the reference group, the life expectancy at age 45 in the highest BRI group (Q5) was significantly reduced by 2.2 years. Previous studies have also shown that obesity can significantly shorten life expectancy and accelerate biological aging by reducing the length of leukocyte telomeres (28, 29). Obesity not only significantly increases the risk of death due to CVDs but also reduces life expectancy (30–32). Moreover, the impact of obesity on life expectancy is not only reflected in the increase of CVDs but also includes negative effects on overall health, such as the shortening of healthy life expectancy. This means that obese individuals may experience more health issues and chronic diseases later in life (33).

Regarding gender differences, this study found that males at higher BRI levels (Q4 and Q5) exhibited a more pronounced increase in death risk (HR: 1.24–1.56), and the loss of life expectancy was more significant (up to 3.8 years). In contrast, the risk pattern for females was more complex, with moderate to high levels (Q4) showing a protective effect. Mechanistically, these sex disparities likely reflect differential visceral fat accumulation patterns and hormonal influences. Visceral adipose tissue, which has higher metabolic activity and pro-inflammatory potential than subcutaneous fat, may mediate much of the BRI-mortality association through chronic low-grade inflammation, insulin resistance, and endothelial dysfunction—pathways amplified by androgens in males but mitigated by estrogen's anti-inflammatory and vasoprotective effects in pre-menopausal females. This significant gender difference may stem from several factors. Firstly, there are inherent differences in body fat distribution patterns between males and females. Female fat is mainly stored in subcutaneous tissue, while males tend to accumulate visceral fat, which is more closely associated with cardiometabolic risks (34–38). Secondly, estrogen has multiple protective effects, including improving insulin sensitivity, regulating lipid metabolism, and anti-inflammatory properties. This hormonal advantage may buffer the adverse effects of higher body fat levels in females (39). Thirdly, females demonstrate better adaptability in energy metabolism and oxidative stress response, with their adipose tissue exhibiting stronger angiogenic capability and a lesser inflammatory response (40). Lastly, at similar BRI levels, females generally maintain better lifestyles (such as diet quality and physical activity), and these behavioral differences may contribute to better metabolic health status.

Our study also revealed that high BRI is associated with multiple unfavorable lifestyle factors and metabolic abnormalities, including lower physical activity levels (only 100.78 min in the Q5 group), poorer diet quality (with a high proportion of low HEI-2015 scores reaching 78.28%), and a higher incidence of cardiometabolic diseases. These findings underscore the critical role of behavioral interventions in reducing BRI and improving outcomes. Specifically, targeted lifestyle modifications—including increasing physical activity to ≥150 min/week, adopting Mediterranean or DASH dietary patterns to improve HEI scores, and implementing structured weight management programs—represent first-line strategies that could shift patients from high-risk Q5 to lower-risk categories, potentially translating into substantial life expectancy gains. Our findings align with previous studies, demonstrating that unhealthy lifestyles and obesity jointly exert a negative impact on life expectancy trends (41–43). There is considerable variation in the association between BRI and death risk among different populations, as indicated by subgroup analysis. In the population aged ≤65 years, the highest BRI group experienced a 40% rise in the likelihood of death (HR: 1.40), and the risk for current smokers rose to 73% (HR: 1.73). This risk stratification provides an important basis for individualized risk assessment in clinical practice.

Beyond behavioral interventions, the pronounced mortality risk and life expectancy loss associated with elevated BRI suggest a potential role for pharmacological approaches in high-risk individuals. Emerging weight-loss medications, particularly GLP-1 receptor agonists (e.g., semaglutide, tirzepatide), have demonstrated remarkable efficacy in reducing visceral adiposity and improving cardiometabolic parameters. Importantly, existing studies have shown that both GLP-1 receptor agonists and SGLT2 inhibitors can significantly reduce all-cause mortality and cardiovascular events in patients with type 2 diabetes by lowering blood pressure and improving cardiovascular health, with observational data further suggesting that combination therapy offers greater advantages in reducing MACE, heart failure hospitalizations, and all-cause mortality (44–46). Future studies should evaluate whether these pharmacological agents can lower BRI below our identified thresholds (5.13 for all-cause, 5.56 for CVD mortality) and whether such reductions directly correspond to mortality benefits and life expectancy extension. Additionally, SGLT2 inhibitors and newer dual/triple agonists targeting multiple metabolic pathways warrant investigation for their BRI-modifying potential.

Taken together, these results suggest that BRI may serve as a useful, non-invasive metric for risk refinement in MetS populations. Our findings may help identify high-risk patients (especially males and those ≤65 years) who could benefit from more intensive lifestyle counseling or pharmacotherapy evaluation. The quantifiable life expectancy impact may provide a valuable communication tool to motivate behavior change and monitor treatment response. While our observational data cannot establish causality, they provide preliminary evidence that could inform future research into sex-specific BRI screening cutoffs and risk-stratified intervention algorithms, potentially contributing to more personalized cardiometabolic risk management strategies.

This study possesses several strengths: it employs a long time span (1999–2018) NHANES database with a large sample size (*n* = 19,535); it specifically targets the MetS patient population, enhancing its clinical reference value; it innovatively correlates BRI directly with life expectancy, quantifying the specific number of years lost; and it utilizes a comprehensive multiple imputation method to address missing values, considering adjustments for multiple confounding factors. However, there are still some limitations to this study. Firstly, as an observational study based on the NHANES database, it cannot determine the causal relationship between BRI and death risk. Secondly, This analysis relied on baseline BRI measurements, which, while providing a snapshot of association, cannot account for intra-individual fluctuations in BRI over time or capture the influence of BRI trajectories on life expectancy. Future studies incorporating repeated BRI assessments and employing longitudinal trajectory analysis to examine how stable, increasing, or decreasing BRI patterns relate to differential mortality risk and life expectancy would be of greater clinical value. Integrating biomarkers—such as high-sensitivity C-reactive protein (hs-CRP), adiponectin, leptin, and insulin resistance markers—could help elucidate the biological pathways linking BRI to mortality and potentially identify novel therapeutic targets. Such an approach would more accurately reflect the dynamic nature of adiposity and its long-term health consequences, strengthen causal inference, and offer insights into critical windows for intervention. Furthermore, although the study adjusted for multiple confounding factors, there may still be potential confounders that were not collected or considered, and some self-reported covariates may be subject to reporting bias. Additionally, the study's focus on MetS patients may limit the generalizability of the results. Finally, We acknowledge that approximately 44.5% of the original cohort was excluded, primarily due to ineligible mortality follow-up status and participants who did not complete both the interview and MEC examination. This exclusion was necessary to ensure the reliability of mortality outcomes and the availability of objectively measured BRI data. While we applied appropriate sampling weights to enhance representativeness, the potential for selection bias remains an important limitation. Future research should consider conducting prospective cohort studies, performing multiple BRI measurements, collecting more detailed cause of death classification information, and exploring the biological mechanisms underlying the tie between BRI and the probability of mortality to provide stronger guidance for clinical practice.

## 5 Conclusion

In conclusion, our findings indicate that although BRI did not demonstrate superior predictive performance for mortality compared to BMI in this population, an elevated BRI is independently associated with increased mortality risk and reduced life expectancy in patients with metabolic syndrome, with a more pronounced effect in males. These results support the clinical utility of monitoring BRI alongside traditional indicators like BMI for risk assessment in this population, and suggest its potential integration into cardiometabolic risk calculators to refine stratification and guide management.

## Data Availability

Publicly available datasets were analyzed in this study. This data can be found here: the datasets produced and analyzed in this research are accessible in the NHANES database (https://wwwn.cdc.gov/nchs/nhanes/default.aspx).

## References

[1] Choudhary P Singh VK Dixit A. 2D-bio-FETs for sensitive detection of cardiovascular diseases. J Phys Condens Matter. (2024) 36:413004. 10.1088/1361-648x/ad5ee9 38959912

[2] Gurka MJ Filipp SL Musani SK Sims M DeBoer MD. Use of BMI as the marker of adiposity in a metabolic syndrome severity score: derivation and validation in predicting long-term disease outcomes. Metab Clin Exp. (2018) 83:68–74. 10.1016/j.metabol.2018.01.015 29410278 PMC5960618

[3] Li Z Fan C Huang J Chen Z Yu X Qian J. Non-linear relationship between the body roundness index and metabolic syndrome: data from national health and nutrition examination survey (NHANES) 1999–2018. Br J Nutr. (2024) 131:1852–9. 10.1017/S0007114524000357 38356387

[4] Huh JH Lee JH Moon JS Sung KC Kim JY Kang DR. Metabolic syndrome severity score in Korean adults: analysis of the 2010–2015 Korea national health and nutrition examination survey. J Korean Med Sci. (2019) 34:e48. 10.3346/jkms.2019.34.e48 30787681 PMC6374550

[5] Matacchione G Perugini J Di Mercurio E Sabbatinelli J Prattichizzo F Senzacqua M Senescent macrophages in the human adipose tissue as a source of inflammaging. Geroscience. (2022) 44:1941–60. 10.1007/s11357-022-00536-0 35247131 PMC9616990

[6] Moon HU Ha KH Han SJ Kim HJ Kim DJ. The association of adiponectin and visceral fat with insulin resistance and β-cell dysfunction. J Korean Med Sci. (2019) 34:e7. 10.3346/jkms.2019.34.e7 30618514 PMC6318440

[7] Eldakhakhny B Bima A Alamoudi AA Alnami A Abo-Elkhair SM Sakr H The role of low-carbohydrate, high-fat diet in modulating autophagy and endoplasmic reticulum stress in aortic endothelial dysfunction of metabolic syndrome animal model. Front Nutr. (2024) 11:1467719. 10.3389/fnut.2024.1467719 39610878 PMC11603365

[8] Oyerinde AS Selvaraju V Babu JR Geetha T. Potential role of oxidative stress in the production of volatile organic compounds in obesity. Antioxidants (Basel). (2023) 12:129. 10.3390/antiox12010129 36670991 PMC9854577

[9] Saponaro C Sabatini S Gaggini M Carli F Rosso C Positano V Adipose tissue dysfunction and visceral fat are associated with hepatic insulin resistance and severity of NASH even in lean individuals. Liver Int. (2022) 42:2418–27. 10.1111/liv.15377 35900229

[10] Pan H Lin S. Association of hemoglobin, albumin, lymphocyte, and platelet score with risk of cerebrovascular, cardiovascular, and all-cause mortality in the general population: results from the NHANES 1999–2018. Front Endocrinol. (2023) 14:1173399. 10.3389/fendo.2023.1173399 PMC10328756 37424853

[11] Adegoke O Ozoh OB Odeniyi IA Bello BT Akinkugbe AO Ojo OO Prevalence of obesity and an interrogation of the correlation between anthropometric indices and blood pressures in urban Lagos, Nigeria. Sci Rep. (2021) 11:3522. 10.1038/s41598-021-83055-w 33568712 PMC7876118

[12] Liu W Song S Zhang H An C. A comparative analysis of anthropometric indices for predicting obstructive sleep apnea among American Adults. Sci Rep. (2024) 14(1):29578. 10.1038/s41598-024-81191-7 39609575 PMC11604657

[13] Wu LD Kong C Shi Y Zhang J Chen SL. Associations between novel anthropometric measures and the prevalence of hypertension among 45,853 adults: a cross-sectional study. Front Cardiovasc Med. (2022) 9:1050654. 10.3389/fcvm.2022.1050654 36407444 PMC9669705

[14] Murakami K Fujii R Tsuboi Y Okumiyama H Umematsu R Suzuki K. Body roundness index and all-cause and CVD mortality: findings from Japanese adults and meta-analysis. J Nutr. (2025) 155(12):4549–55. 10.1016/j.tjnut.2025.09.031 41022153

[15] Zhou D Liu X Huang Y Feng Y. A nonlinear association between body roundness index and all-cause mortality and cardiovascular mortality in general population. Public Health Nutr. (2022) 25:3008–15. 10.1017/S1368980022001768 35983642 PMC9991644

[16] Zhang X Ma N Lin Q Chen K Zheng F Wu J Body roundness index and all-cause mortality among US adults. JAMA Netw Open (2024) 7:e2415051. 10.1001/jamanetworkopen.2024.15051 38837158 PMC11154161

[17] Wu M Yu X Xu L Wu S Tian Y. Associations of longitudinal trajectories in body roundness index with mortality and cardiovascular outcomes: a cohort study. Am J Clin Nutr. (2022) 115:671–8. 10.1093/ajcn/nqab412 34918019

[18] Liu Y Liu XC Guan H Zhang S Zhu Q Fu X Body roundness Index is a superior obesity index in predicting diabetes risk among hypertensive patients: a prospective cohort study in China. Front Cardiovasc Med. (2021) 8:736073. 10.3389/fcvm.2021.736073 34869638 PMC8638826

[19] Bai Q Chen H Gao Z Li B Liu S Dong W Advanced prediction of heart failure risk in elderly diabetic and hypertensive patients using nine machine learning models and novel composite indices: insights from NHANES 2003–2016. Eur J Prev Cardiol. (2025):zwaf081. 10.1093/eurjpc/zwaf081 40036490

[20] Wang P Fan Y Gao H Wang B. Body roundness index as a predictor of all-cause and cardiovascular mortality in patients with diabetes and prediabetes. Diabetes Res Clin Pract. (2025) 219:111958. 10.1016/j.diabres.2024.111958 39675484

[21] Tao L Miao L Guo Y-J Liu Y-L Xiao L-H Yang Z-J. Associations of body roundness index with cardiovascular and all-cause mortality: NHANES 2001–2018. J Hum Hypertens (2023) 38:120–7. 10.1038/s41371-023-00864-4 37752175

[22] Ding Z Li W Qi H Fang T Zhu Q Qu X The L-shaped association between body roundness index and all-cause mortality in osteoporotic patients: a cohort study based on NHANES data. Front Nutr. (2025) 12:1538766. 10.3389/fnut.2025.1538766 39902313 PMC11788163

[23] Ding J Chen X Shi Z Bai K Shi S. Association of body roundness index and its trajectories with all-cause and cardiovascular mortality among a Chinese middle-aged and older population: a retrospective cohort study. Front Public Health. (2023) 11:1107158. 10.3389/fpubh.2023.1107158 37033022 PMC10076882

[24] Liu L Feng J Zhang G Yuan X Li F Yang T Visceral adipose tissue is more strongly associated with insulin resistance than subcutaneous adipose tissue in Chinese subjects with pre-diabetes. Curr Med Res Opin. (2017) 34:123–9. 10.1080/03007995.2017.1364226 28776439

[25] Zeng D Zeng Q Li S Lu J Cheng N. Evaluating body roundness index and systemic immune inflammation index for mortality prediction in MAFLD patients. Sci Rep. (2025) 15:330. 10.1038/s41598-024-83324-4 39747385 PMC11695853

[26] Al-Shami I Alkhalidy H Alnaser K Mukattash TL Al Hourani H Alzboun T Assessing metabolic syndrome prediction quality using seven anthropometric indices among Jordanian adults: a cross-sectional study. Sci Rep (2022) 12:21043. 10.1038/s41598-022-25005-8 36473903 PMC9727133

[27] Mansoori A Seifi N Vahabzadeh R Hajiabadi F Mood MH Harimi M The relationship between anthropometric indices and the presence of hypertension in an Iranian population sample using data mining algorithms. J Hum Hypertens. (2023) 38:277–85. 10.1038/s41371-023-00877-z 38040904

[28] Brønnum-Hansen H Davidsen M Andersen I. Impact of the association between education and obesity on diabetes-free life expectancy. Eur J Public Health. (2023) 33:968–73. 10.1093/eurpub/ckad153 37615997 PMC10710352

[29] Zhang Y Xu Z Yang Y Cao S Lyu S Duan W. Association between weight change and leukocyte telomere length in U.S. adults. Front Endocrinol. (2021) 12:650988. 10.3389/fendo.2021.650988 PMC8355991 34393992

[30] Kitahara CM Flint AJ Berrington de Gonzalez A Bernstein L Brotzman M MacInnis RJ Association between class III obesity (BMI of 40–59 kg/m2) and mortality: a pooled analysis of 20 prospective studies. PLoS Med (2014) 11:e1001673. 10.1371/journal.pmed.1001673 25003901 PMC4087039

[31] Kotsis V Tsioufis K Antza C Seravalle G Coca A Sierra C Obesity and cardiovascular risk: a call for action from the European society of hypertension working group of obesity, diabetes and the high-risk patient and European association for the study of obesity: part B: obesity-induced cardiovascular disease, early prevention strategies and future research directions. J Hypertens (2018) 36:1441–55. 10.1097/HJH.0000000000001731 29652731

[32] Vidra N Trias-Llimós S Janssen F. Impact of obesity on life expectancy among different European countries: secondary analysis of population-level data over the 1975–2012 period. BMJ Open. (2019) 9:e028086. 10.1136/bmjopen-2018-028086 31371290 PMC6678519

[33] Stenholm S Head J Aalto V Kivimäki M Kawachi I Zins M Body mass index as a predictor of healthy and disease-free life expectancy between ages 50 and 75: a multicohort study. Int J Obes. (2017) 41:769–75. 10.1038/ijo.2017.29 PMC5418561 28138135

[34] Yang Y Xie M Yuan S Zeng Y Dong Y Wang Z Sex differences in the associations between adiposity distribution and cardiometabolic risk factors in overweight or obese individuals: a cross-sectional study. BMC Public Health (2021) 21:1232. 10.1186/s12889-021-11316-4 34174845 PMC8234731

[35] Gealekman O Guseva N Hartigan C Apotheker S Gorgoglione M Gurav K Depot-specific differences and insufficient subcutaneous adipose tissue angiogenesis in human obesity. Circulation. (2011) 123(2):186–94. 10.1161/CIRCULATIONAHA.110.970145 21200001 PMC3334340

[36] Figueroa AL Takx RAP MacNabb MH Abdelbaky A Lavender ZR Kaplan RS Relationship between measures of adiposity, arterial inflammation, and subsequent cardiovascular events. Circ Cardiovasc Imaging (2016) 9:e004043. 10.1161/CIRCIMAGING.115.004043 27072302 PMC5036397

[37] Sato F Maeda N Yamada T Namazui H Fukuda S Natsukawa T Association of epicardial, visceral, and subcutaneous fat with cardiometabolic diseases. Circ J. (2018) 82:502–8. 10.1253/circj.CJ-17-0820 28954947

[38] Lee JJ Pedley A Hoffmann U Massaro JM Levy D Long MT. Visceral and intrahepatic fat are associated with cardiometabolic risk factors above other ectopic fat depots: the Framingham heart study. Am J Med (2018) 131:684–692.e12. 10.1016/j.amjmed.2018.02.002 29518370 PMC5964004

[39] Mauvais-Jarvis F Lindsey SH. Metabolic benefits afforded by estradiol and testosterone in both sexes: clinical considerations. J Clin Invest (2024) 134:e180073. 10.1172/JCI180073 39225098 PMC11364390

[40] Moreau KL Ozemek C. Vascular adaptations to habitual exercise in older adults: time for the sex talk. Exercise Sport Sci Rev. (2017) 45:116–23. 10.1249/JES.0000000000000104 PMC5357172 28092297

[41] Janssen F Trias-Llimós S Kunst AE. The combined impact of smoking, obesity and alcohol on life-expectancy trends in Europe. Int J Epidemiol. (2021) 50:931–41. 10.1093/ije/dyaa273 33432332 PMC8271206

[42] Stenholm S Head J Kivimäki M Kawachi I Aalto V Zins M Smoking, physical inactivity and obesity as predictors of healthy and disease-free life expectancy between ages 50 and 75: a multicohort study. Int J Epidemiol (2016) 45:1260–70. 10.1093/ije/dyw126 27488415 PMC6937009

[43] Rahman MM Jagger C Leigh L Holliday E Princehorn E Loxton D The impact of education and lifestyle factors on disability-free life expectancy from mid-life to older age: a multi-cohort study. Int J Public Health. (2022) 67:1605045. 10.3389/ijph.2022.1605045 36046258 PMC9421499

[44] Colombijn JMT de Leijer JF Visseren FLJ Verhaar MC van Raalte DH Sattar N Effectiveness and safety of combining SGLT2 inhibitors and GLP-1 receptor agonists in individuals with type 2 diabetes: a systematic review and meta-analysis of cohort studies. Diabetologia. (2026) 69(1):36–49. 10.1007/s00125-025-06565-6 41117973 PMC12686040

[45] Diallo A Carlos-Bolumbu M Galtier F. Blood pressure-lowering effects of SGLT2 inhibitors and GLP-1 receptor agonists for preventing of cardiovascular events and death in type 2 diabetes: a systematic review and meta-analysis. Acta Diabetol. (2023) 60:1651–62. 10.1007/s00592-023-02154-4 37439858

[46] Scheen AJ. Cardiovascular and renal effects of the combination therapy of a GLP-1 receptor agonist and an SGLT2 inhibitor in observational real-life studies. Diabetes Metab. (2025) 51:101594. 10.1016/j.diabet.2024.101594 39608670

