# Supplementary material for: Relationship between body roundness index and mortality rates and life expectancy in populations with metabolic syndrome
Source: Front Cardiovasc Med. 2026 Jan 5;12:1608436. doi: 10.3389/fcvm.2025.1608436 (PMC12812642; doi:10.3389/fcvm.2025.1608436)
Supplement: Supplementary file 1 [file Table1.docx]

Supplementary Material

**
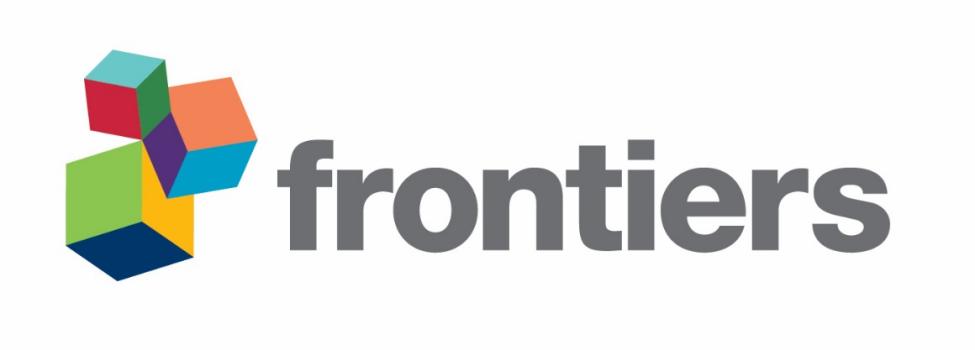
**

**Supplementary Figure 1.** Study Population Flowchart


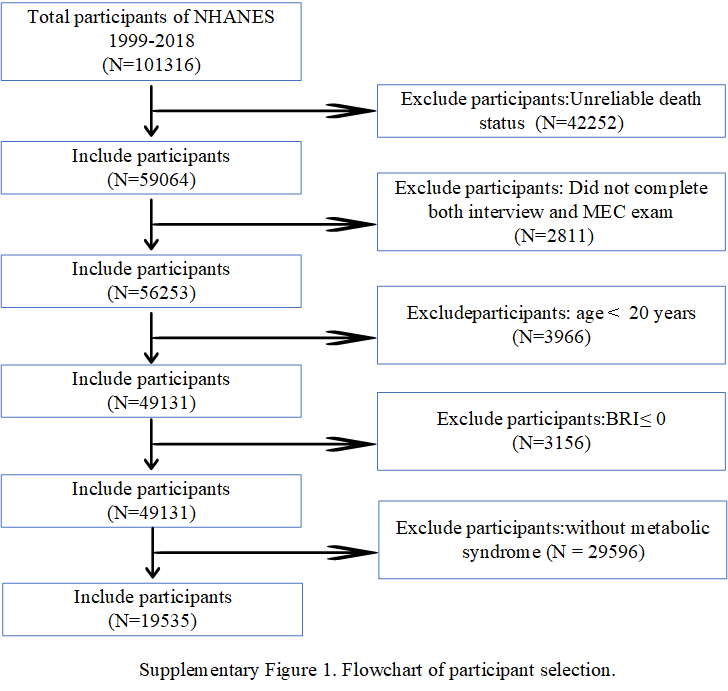


**Supplementary Figure 2.** Nonlinear relationship between BRI and all-cause mortality and cardiovascular mortality using curve fitting analysis.


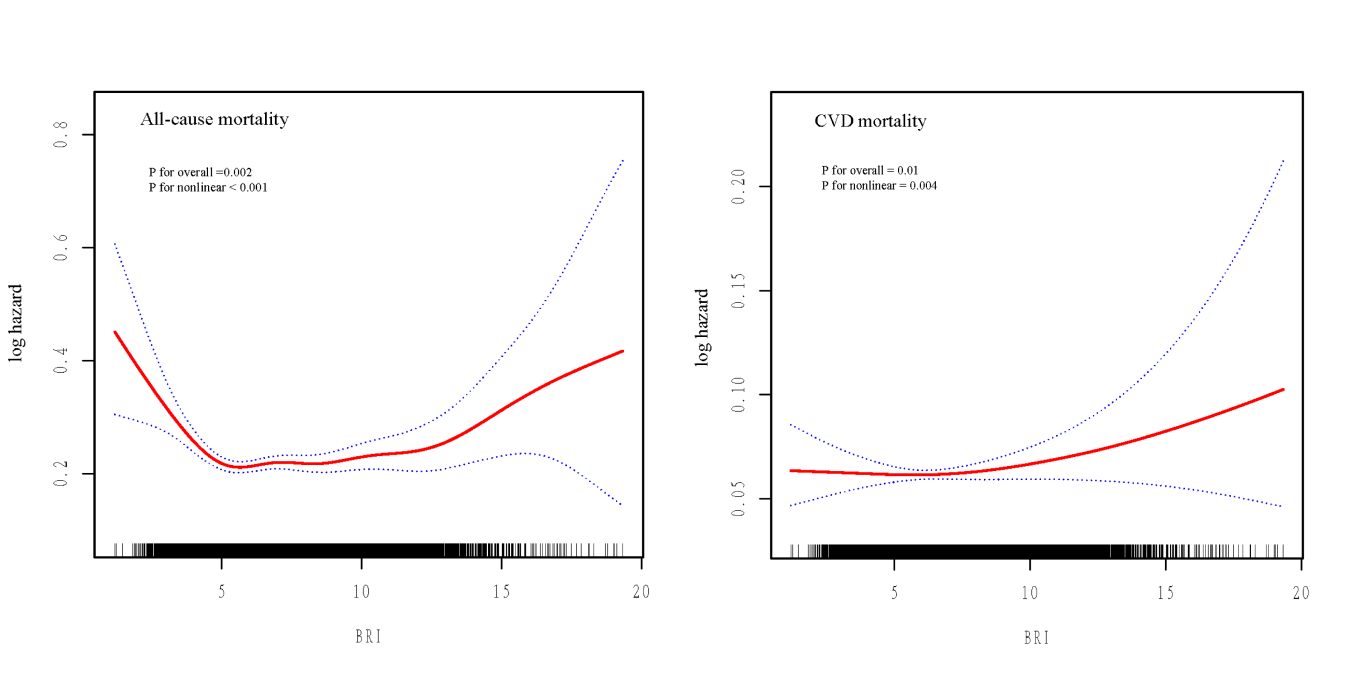


Adjusted for sex, age(continuous), race/ethnicity, education, Family income-poverty ratio, Drinking status, Smoking status, Family history of diabetes, Marital status, HEI-2015 score(continuous), Leisure-time physical activity(continuous), and Other comorbidities(CVD, Hyperuricemia).

**Supplementary Figure 3** Receiver operating characteristic curves for BRI and BMI predicting all-cause mortality


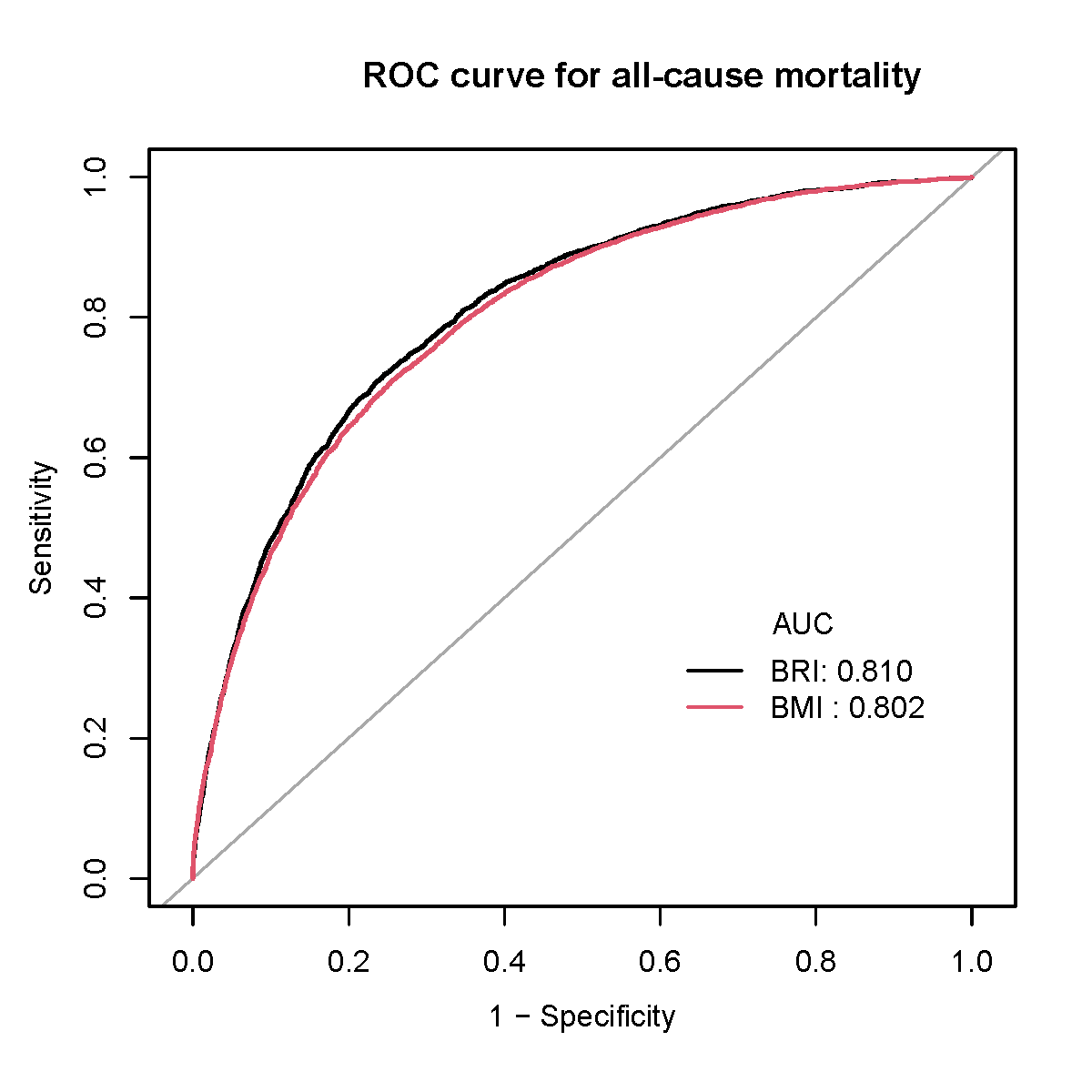


**Supplementary Table 1**. Hazard ratios of BRI for mortality risk in metabolic syndrome(exclude individuals who died within the 2 years prior to follow-up)

| **All-cause mortality** | Event | Crude Model HR (95% CI)a | **P** | Model 1 HR (95% CI)a | **P** | Model 2 HR  (95% CI)a | **P** |
| --- | --- | --- | --- | --- | --- | --- | --- |
| BRI |  | 1.03(1.01,1.05) | 0.01 | 1.08(1.05,1.10) | <0.001 | 1.05(1.02,1.08) | 0.0002 |
| BRI(Quartile) |  |  |  |  |  |  |  |
| Q1(1.19-5.02) | 743 | 0.84 (0.74-0.95) | 0.01 | 0.97 (0.86-1.09) | 0.57 | 1.01 (0.89-1.15) | 0.8608 |
| Q2(5.02-5.88) | 696 | 0.79 (0.68-0.90) | <0.001 | 0.84 (0.74-0.96) | 0.01 | 0.89 (0.78-1.02) | 0.085 |
| Q3(5.88-6.81) | 768 | 1 (reference) |  | 1 (reference) |  | 1 (reference) |  |
| Q4(6.81-8.22) | 736 | 0.96 (0.86-1.08) | 0.53 | 1.04 (0.92-1.18) | 0.51 | 1.01 (0.90-1.15) | 0.8237 |
| Q5(8.22-23.48) | 652 | 1.04 (0.89-1.21) | 0.61 | 1.40 (1.20-1.64) | <0.001 | 1.28 (1.09-1.51) | 0.0024 |
| P for trend |  | <0.001 |  |  | <0.001 |  | <0.001 |
| **CVD mortality** |  |  |  |  |  |  |  |
| BRI |  | 1.06(1.04,1.10) | <0.001 | 1.13(1.09,1.18) | <0.001 | 1.10 (1.05,1.14) | < 0.0001 |
| BRI(Quartile) |  |  |  |  |  |  |  |
| Q1(1.19-5.02) | 201 | 0.76 (0.62-0.93) | 0.01 | 0.89 (0.73-1.08) | 0.23 | 0.92 (0.75-1.13) | 0.42 |
| Q2(5.02-5.88) | 181 | 0.80 (0.65-0.99) | 0.04 | 0.87 (0.71-1.06) | 0.15 | 0.92 (0.75-1.13) | 0.42 |
| Q3(5.88-6.81) | 218 | 1 (reference) |  | 1 (reference) |  | 1 (reference) |  |
| Q4(6.81-8.22) | 205 | 1.05 (0.86-1.28) | 0.64 | 1.16 (0.95-1.41) | 0.15 | 1.10 (0.90-1.35) | 0.34 |
| Q5(8.22-23.48) | 186 | 1.19 (0.97-1.46) | 0.09 | 1.72 (1.38-2.13) | <0.001 | 1.46 (1.16-1.82) | 0.001 |
| P for trend |  | <0.001 |  |  | <0.001 |  | <0.001 |

Crude Model: Unadjusted results

Model 1: Results adjusted for age and sex;

Model 2:Results adjusted for sex, age(continuous), race/ethnicity, education, family income-poverty ratio, drinking status, smoking status, family history of diabetes, marital status, HEI-2015 score(continuous), leisure-time physical activity(continuous), and other comorbidities(CVD, hyperuricemia).

^a^ sampling weights were considered in analyses

**Supplementary table 2.** hazard ratios of BRI for mortality risk in metabolic syndrome(exclude participants with cardiovascular disease or cancer during the baseline survey)

| **All-cause mortality** | Event | Crude Model HR (95% CI)a | **P** | Model 1 HR (95% CI)a | **P** | Model 2 HR  (95% CI)^a^ | **P** |
| --- | --- | --- | --- | --- | --- | --- | --- |
| BRI |  | 1.04(1.01,1.07) | 0.004 | 1.08(1.04,1.11) | <0.001 | 1.06(1.03,1.10) | <0.001 |
| BRI(Quartile) |  |  |  |  |  |  |  |
| Q1(1.19-5.02) | 461 | 0.79 (0.67-0.92) | 0.002 | 0.95 (0.82-1.11) | 0.52 | 1.01 (0.87-1.18) | 0.88 |
| Q2(5.02-5.88) | 436 | 0.72 (0.61-0.85) | <0.001 | 0.78 (0.67-0.92) | 0.004 | 0.84 (0.71-0.99) | 0.04 |
| Q3(5.88-6.81) | 485 | 1 (reference) |  | 1 (reference) |  | 1 (reference) |  |
| Q4(6.81-8.22) | 454 | 0.88 (0.76-1.02) | 0.08 | 0.94 (0.81-1.09) | 0.38 | 0.93 (0.80-1.08) | 0.37 |
| Q5(8.22-23.48) | 416 | 1.06 (0.89-1.27) | 0.52 | 1.44 (1.19-1.74) | <0.001 | 1.39 (1.14-1.68) | <0.001 |
| P for trend |  |  | <0.001 |  | <0.001 |  | <0.001 |
| **CVD mortality** |  |  |  |  |  |  |  |
| BRI |  | 1.10(1.05,1.14) | <0.001 | 1.17(1.11,1.23) | <0.001 | 1.13 (1.07,1.20) | < 0.0001 |
| BRI(Quartile) |  |  |  |  |  |  |  |
| Q1(1.19-5.02) | 112 | 0.76 (0.63-0.93) | 0.01 | 0.83 (0.63-1.09) | 0.18 | 0.91 (0.67-1.23) | 0.53 |
| Q2(5.02-5.88) | 98 | 0.76 (0.61-0.94) | 0.01 | 0.78 (0.56-1.07) | 0.12 | 0.84 (0.62-1.16) | 0.29 |
| Q3(5.88-6.81) | 121 | 1 (reference) |  | 1 (reference) |  | 1 (reference) |  |
| Q4(6.81-8.22) | 117 | 0.99 (0.81-1.22) | 0.95 | 1.01 (0.75-1.35) | 0.97 | 1.00 (0.74-1.33) | 0.98 |
| Q5(8.22-23.48) | 113 | 1.17 (0.96-1.43) | 0.12 | 1.96 (1.46-2.61) | <0.001 | 1.81 (1.36-2.42) | <0.001 |
| P for trend |  |  | <0.001 |  | <0.001 |  | <0.001 |

Crude Model: Unadjusted results

Model 1: Results adjusted for age and sex;

Model 2:Results adjusted for sex, age(continuous), race/ethnicity, education, family income-poverty ratio, drinking status, smoking status, family history of diabetes, marital status, HEI-2015 score(continuous), leisure-time physical activity(continuous), and other comorbidities(CVD, hyperuricemia).

^a^ sampling weights were considered in analyses

**Supplementary table 3.** Hazard ratios of BRI for mortality risk in metabolic syndrome(further adjustments to the NHANES survey cycle)

|  | **Event** | **All-cause mortality** | **P** | **CVD mortality** | **P** |
| --- | --- | --- | --- | --- | --- |
|  |  | Model 2 HR  (95% CI)a |  | Model 2 HR (95% CI)a |  |
| BRI |  | 1.05 (1.03,1.07) | <0.001 | 1.09 (1.05,1.14) | < 0.001 |
| BRI(Quartile) |  |  |  |  |  |
| Q1(1.19-5.02) | 794 | 1.01 (0.89-1.14) | 0.91 | 0.91 (0.74-1.12) | 0.37 |
| Q2(5.02-5.88) | 870 | 0.89 (0.78-1.02) | 0.09 | 0.86 (0.70-1.07) | 0.19 |
| Q3(5.88-6.81) | 855 | 1 (reference) |  | 1 (reference) |  |
| Q4(6.81-8.22) | 833 | 0.98 (0.87-1.11) | 0.78 | 1.03 (0.84-1.26) | 0.77 |
| Q5(8.22-23.48) | 753 | 1.29 (1.12-1.48) | < 0.001 | 1.40 (1.13-1.73) | 0.002 |
| P for trend |  |  | < 0.001 |  | < 0.001 |

After adjusting for sex, age, race/ethnicity, education, family income-to-poverty ratio, drinking and smoking status, family history of diabetes, marital status, diet quality, physical activity, and comorbidities, we additionally controlled for NHANES survey cycles.

**Supplementary Figure 4** Stratified Analysis Study on the Association Between BRI and Mortality Rates

Note:Stratified analyses were adjusted for covariates including sex, age (continuous), race/ethnicity, education, family income-poverty ratio, drinking status, smoking status, family history of diabetes, marital status, HEI-2015 score (continuous), leisure-time physical activity (continuous), and other comorbidities (cardiovascular disease, hyperuricemia).

**Supplementary Figure 5**. Expected Lifespan at Age 55: stratified analysis by body roundness index in totalpopulation, women, and men


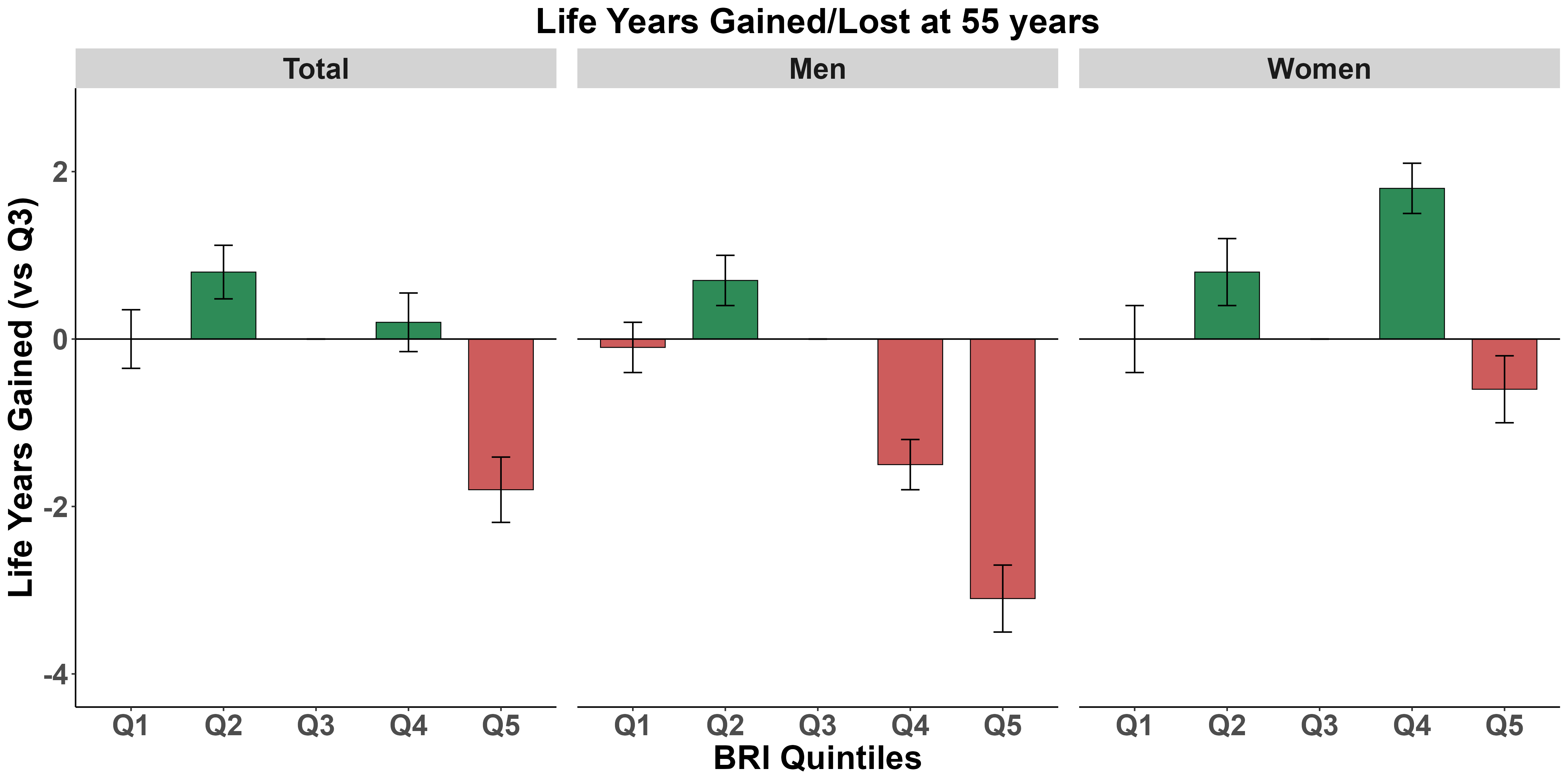


Green indicates lifespan gain, while red indicates lifespan loss

**Supplementary Figure 6**. Expected Lifespan at Age 65: stratified analysis by body roundness index in totalpopulation, women, and men


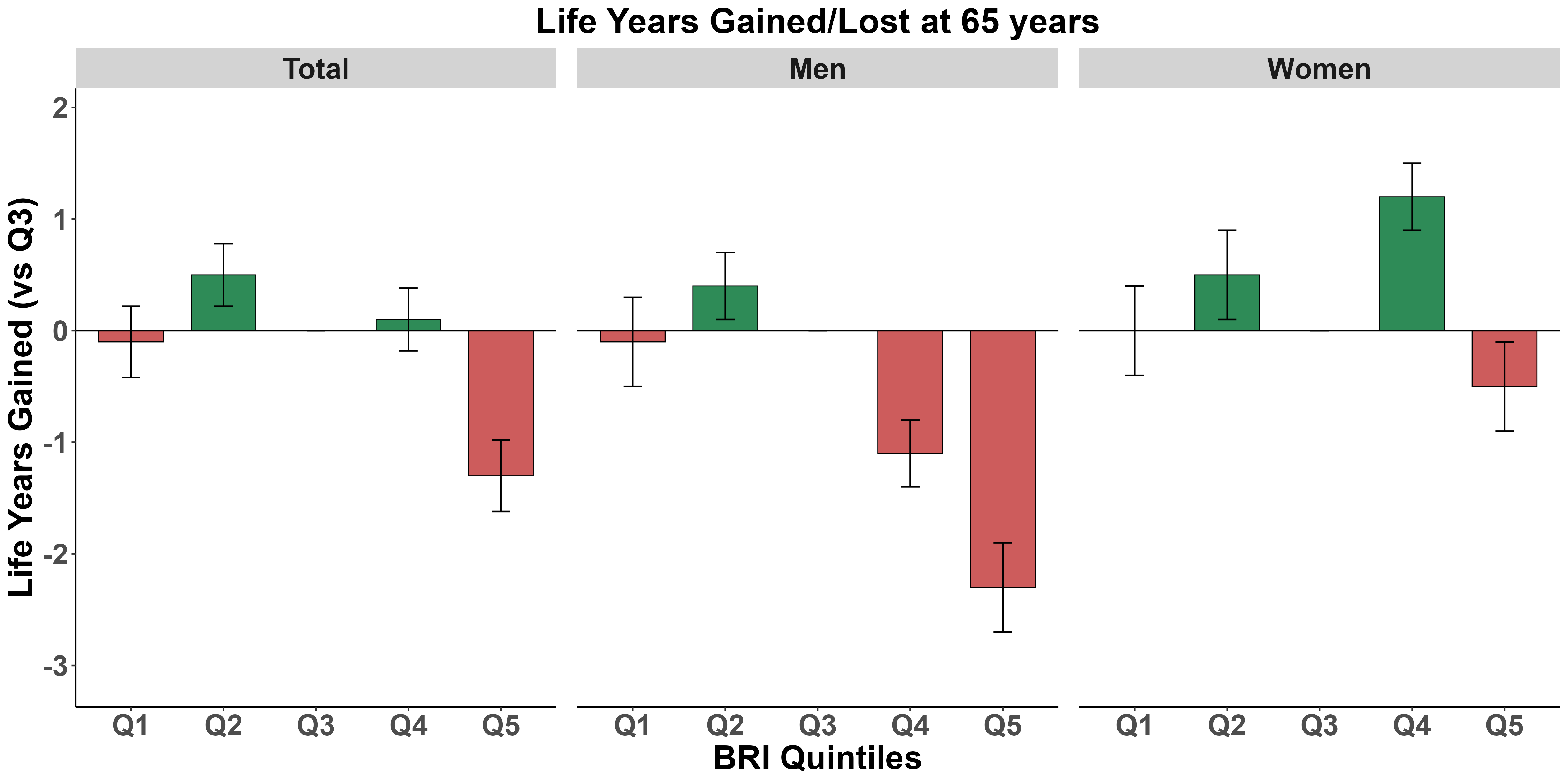


Green indicates lifespan gain, while red indicates lifespan loss

**Supplementary Tables 4.** Missing variables in the study population

| **Variable Name** | **Number of Missing Values** | **Missing Percentage (%)** |
| --- | --- | --- |
| HEI-2015 score | 26 | 0.13 |
| BMI | 847 | 4.53 |
| Age | 42 | 0.21 |
| Education level | 23 | 0.12 |
| Income | 1747 | 9 |
| Marital status | 173 | 0.89 |
| Smoking | 15 | 0.08 |
| Drinking status | 2455 | 12.59 |

**Supplementary Tables 5** Hazard ratios for BRI and mortality in persons without metabolic syndrome

| **All-cause mortality** | **Event** | **Crude Model HR**  **(95% CI)a** | **P** | **Model 1 HR**  **(95% CI)a** | **P** | **Model 2 HR**  **(95% CI)^a^** | **P** |
| --- | --- | --- | --- | --- | --- | --- | --- |
| BRI |  | 1.09(1.07,1.11) | <0.001 | 1.00(0.97,1.02) | 0.77 | 0.99 (0.96,1.01) | 0.33 |
| BRI(Quartile) |  |  |  |  |  |  |  |
| Q1(<3.04) | 475 | 0.67 (0.57-0.77) | <0.001 | 1.47 (1.26-1.71) | <0.001 | 1.28 (1.09-1.51) | 0.003 |
| Q2(4.04-3.86) | 568 | 0.79 (0.69-0.89) | <0.001 | 1.07 (0.94-1.22) | 0.29 | 1.04 (0.92-1.18) | 0.54 |
| Q3(3.86-4.64) | 674 | 1 (reference) |  | 1 (reference) |  | 1 (reference) |  |
| Q4(4.64-5.82) | 702 | 1.07 (0.95-1.22) | 0.25 | 0.95 (0.84-1.08) | 0.45 | 0.92 (0.81-1.05) | 0.22 |
| Q5(>5.82) | 658 | 1.17 (1.01-1.36) | 0.03 | 1.21 (1.06-1.38) | 0.005 | 1.08 (0.94-1.24) | 0.30 |
| P for trend |  |  | <0.001 |  | 0.01 |  | 0.01 |
| **CVD mortality** |  |  |  |  |  |  |  |
| BRI |  | 1.14(1.10,1.19) | <0.001 | 1.07(0.99,1.14) | 0.07 | 1.05 (0.98,1.12) | 0.19 |
| BRI(Quartile) |  |  |  |  |  |  |  |
| Q1(<3.04) | 83 | 0.48 (0.34-0.66) | <0.001 | 1.22 (0.87-1.72) | 0.24 | 1.05 (0.73-1.52) | 0.78 |
| Q2(4.04-3.86) | 134 | 0.83 (0.63-1.10) | 0.20 | 1.20 (0.92-1.57) | 0.18 | 1.14 (0.86-1.51) | 0.35 |
| Q3(3.86-4.64) | 159 | 1 (reference) |  | 1 (reference) |  | 1 (reference) |  |
| Q4(4.64-5.82) | 176 | 1.12 (0.86-1.46) | 0.39 | 0.98 (0.75-1.28) | 0.88 | 0.95 (0.73-1.24) | 0.71 |
| Q5(>5.82) | 163 | 1.30 (0.99-1.71) | 0.06 | 1.33 (1.03-1.71) | 0.03 | 1.15 (0.89-1.50) | 0.29 |
| P for trend |  |  | <0.001 |  | 0.89 |  | 0.98 |

Crude Model: Unadjusted results

Model 1: Results adjusted for age and sex;

Model 2:Results adjusted for sex, age(continuous), race/ethnicity, education, family income-poverty ratio, drinking status, smoking status, family history of diabetes, marital status, HEI-2015 score(continuous), leisure-time physical activity(continuous), and other comorbidities(CVD, hyperuricemia).

^a^ sampling weights were considered in analyses

**Supplementary Tables 6** Characteristics of the study population by BRI quintile

| **BRI Quintile** | **Total N** | **Events** | **Censored** | **Mean follow-up time (years)** |
| --- | --- | --- | --- | --- |
| Q3 (reference group) | 3,907 | 870 | 3,037 | 8.9 |
| Q1 | 3,908 | 855 | 3,053 | 9.2 |
| Q2 | 3,906 | 794 | 3,112 | 9.2 |
| Q4 | 3,907 | 833 | 3,074 | 8.7 |
| Q5 | 3,907 | 753 | 3,154 | 8.3 |
| ****Total**** | ****19,535**** | ****4,105**** | ****15,430**** | - |

**Supplementary Tables 7** Hazard ratios of BRI for mortality risk in metabolic syndrome

| **CVD mortality** | **event**  **(per 1000 person-years)** | **Crude Model SHR**  **(95% CI)a** | **P** | **Model 1 SHR**  **(95% CI)a** | **P** | **Model 2 SHR**  **(95% CI)^a^** | **P** |
| --- | --- | --- | --- | --- | --- | --- | --- |
| BRI |  | 1.06(1.03,1.09) | <0.001 | 1.13(1.09,1.18) | <0.001 | 1.08 (1.03,1.13) | < 0.001 |
| BRI(quintiles) |  |  |  |  |  |  |  |
| Q1(1.19-5.02) | 229(7.15) | 0.75 (0.62-0.91) | 0.004 | 0.89 (0.73-1.08) | 0.22 | 0.96 (0.77-1.20) | 0.73 |
| Q2(5.02-5.88) | 203(6.29) | 0.74 (0.59-0.91) | 0.01 | 0.80 (0.65-0.99) | 0.04 | 0.88 (0.70-1.11) | 0.27 |
| Q3(5.88-6.81) | 246(7.96) | 1 (reference) |  | 1 (reference) |  | 1 (reference) |  |
| Q4(6.81-8.22) | 231(7.61) | 0.98 (0.80-1.20) | 0.81 | 1.05 (0.86-1.29) | 0.60 | 0.99 (0.81-1.21) | 0.94 |
| Q5(8.22-23.48) | 218(7.44) | 1.15 (0.94-1.41) | 0.18 | 1.64 (1.32-2.05) | <0.001 | 1.35 (1.09-1.68) | 0.007 |
| P for trend |  | <0.001 |  |  | <0.001 |  | <0.001 |

Crude Model: Unadjusted results

Model 1: Results adjusted for age and sex;

Model 2:Results adjusted for sex, age(continuous), race/ethnicity, education, family income-poverty ratio, drinking status, smoking status, family history of diabetes, marital status, HEI-2015 score(continuous), leisure-time physical activity(continuous), and other comorbidities(CVD, hyperuricemia).

^a^ sampling weights were considered in analyses
